# Schistosomiasis in the Philippines: A Comprehensive Review of Epidemiology and Current Control

**DOI:** 10.3390/tropicalmed10020029

**Published:** 2025-01-21

**Authors:** Emmanuel John Tabilin, Darren J. Gray, Mario A. Jiz, Mary Lorraine Mationg, Marianette Inobaya, Eleonor Avenido-Cervantes, Megumi Sato, Marcello Otake Sato, Yasuhito Sako, Yi Mu, Hong You, Matthew Kelly, Pengfei Cai, Catherine A. Gordon

**Affiliations:** 1 Molecular Parasitology Laboratory, QIMR Berghofer Medical Research Institute, Brisbane, QLD 4006, Australia; yi.mu@qimrberghofer.edu.au (Y.M.); hong.you@qimrberghofer.edu.au (H.Y.); pengfei.cai@qimrberghofer.edu.au (P.C.); 2 Faculty of Medicine, The University of Queensland, Brisbane, QLD 4072, Australia; 3 Global Health & Tropical Medicine, QIMR Berghofer Medical Research Institute, Brisbane, QLD 4006, Australia; darren.gray@qimrberghofer.edu.au (D.J.G.); rainemationg@gmail.com (M.L.M.); 4 Don McManus Tropical Health Centre, QIMR Berghofer Medical Research Institute, Brisbane, QLD 4006, Australia; 5 Immunology Department, Research Institute of Tropical Medicine, Manila 1781, Philippines; mario.a.jiz@gmail.com (M.A.J.); elyucervantes@gmail.com (E.A.-C.); 6 Department of Epidemiology and Biostatistics, Research Institute of Tropical Medicine, Manila 1781, Philippines; marianette.inobaya@ritm.gov.ph; 7 Graduate School of Health Sciences, Niigata University, Niigata 951-8518, Japan; satomeg@clg.niigata-u.ac.jp; 8 Division of Global Environment Parasitology, Faculty of Medical Technology, Niigata University of Pharmacy and Medical and Life Sciences, Niigata 956-8603, Japan; marcello@nupals.ac.jp; 9 Division of Parasitology, Department of Infectious Diseases, Asahikawa Medical University, Asahikawa 078-8510, Japan; yasusako@asahikawa-med.ac.jp; 10 National Centre for Epidemiology and Population Health, Australian National University, Canberra, ACT 2601, Australia; matthew.kelly@anu.edu.au

**Keywords:** schistosomiasis, *Schistosoma japonicum*, Philippines, epidemiology

## Abstract

*Schistosomiasis japonica* is an infectious parasitic disease caused by infection with the blood fluke *Schistosoma japonicum*, which is endemic in China, small pockets of Indonesia, and the Philippines. Of the three countries, the prevalence of infection is the highest in the Philippines, despite decades of mass drug administration (MDA). As a zoonosis with 46 potential mammalian definitive hosts and a snail intermediate host, the control and eventual elimination of *S. japonicum* requires management of these animal hosts in addition to new interventions for the human hosts, including health education and water, sanitation, and hygiene (WASH) infrastructure. In this review we examine the status and epidemiology of *S. japonicum* in the Philippines with an overview of the current control program there and what needs to be accomplished in the future to control and eliminate this disease in the country.

## 1. Introduction

Schistosomiasis is a parasitic disease caused by infection of blood flukes of the genus *Schistosoma*. As a neglected tropical disease (NTD), it mostly affects poor rural communities in Asia, the Middle East, Africa, and South America; many of them with limited access to safe water and sanitation [1]. In 2021, the World Health Organization (WHO) estimated that 251.4 million people in 51 countries were at risk with schistosomiasis; more than half of this number are school-aged children [2].

In Asia, *Schistosoma japonicum* is the prevalent species with distribution in the Philippines, the People’s Republic of China, and a small focus of Indonesia. Two more species have more localized distribution in the Southeast Asia: *S. mekongi* in areas in Cambodia and Lao People’s Democratic Republic (Lao PDR) surrounding the Mekong Delta, and *S. malayensis* in Malaysia [3]. Unlike other human schistosomes, Asian schistosomes are zoonotic, mostly infecting domestic mammals [3,4].

**Figure 1:**
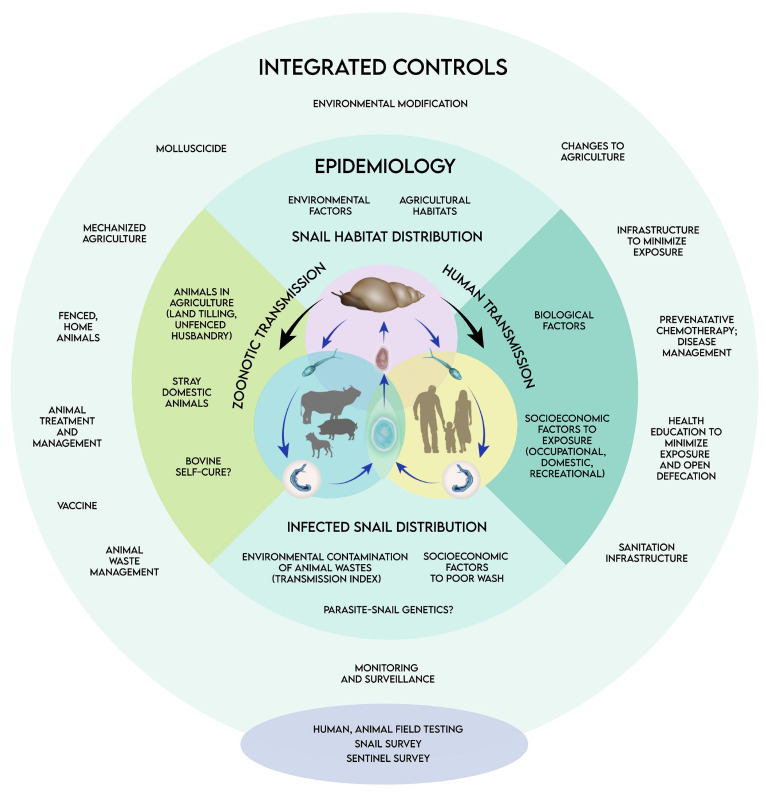
The epidemiology of schistosomiasis in the Philippines is complex with multiple avenues for integrated controls. In the center of the figure is the simplified lifecycle of *S. japonicum,* showing transmission of *S. japonicum* to humans and animals occurring via cercariae released from the snail intermediate host. These cercariae directly penetrate the skin of the mammalian definitive host. They develop into adult worms with males and females pairing up in the portal circulation of the liver, then migrating against the flow of blood in the mesenteric vessels of the bowel to reproduce sexually. The female worm lays eggs which are excreted into the environment by the definitive hosts via the faeces. These eggs hatch upon contact with water into miracidia, which then penetrate the snail host and undergo asexual reproduction. In the next layer, we see the epidemiological factors affecting transmission, and in the outer circle are the proposed integrated control methods to address all epidemiological issues leading to transmission.

Due to its zoonotic nature, the life cycle of *S. japonicum* (Figure 1) is a complex process with both mammalian definitive hosts and molluscan intermediate hosts. Sexual reproduction occurs within the definitive host, while asexual reproduction occurs in the molluscan host [5] (Figure 1).

Nearing elimination in China and three small foci in Indonesia, *S. japonicum* remains highly endemic across communities in the Philippines despite decades of control efforts centered on preventative chemotherapy with praziquantel. Central to this persistence are socioeconomic factors that contribute to the frequent exposure and continuance of the parasite life cycle. This paper reviews the epidemiology of schistosomiasis in the Philippines and the environmental, socioeconomic, and biological factors that determine its distribution and transmission.

## 2. Schistosomiasis in the Philippines

**Figure 2:**
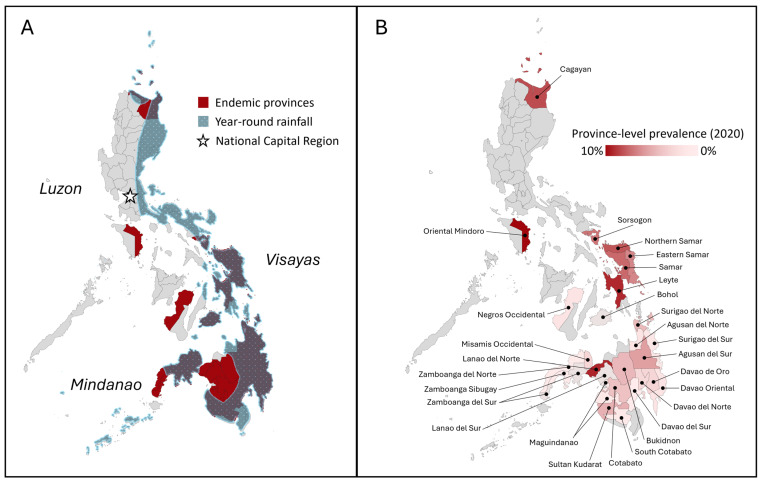
The status of schistosomiasis in the Philippines. (**A**) Philippine map highlighting the schistosomiasis-endemic provinces, as validated by snail surveys [6,7,8,9,10,11,12]. Many of them experience year-round rainfall, illustrating the influence of environmental conditions to schistosomiasis epidemiology [15]. (**B**) The latest province-level prevalence estimates from focal surveys of endemic villages conducted between 2014 and 2018 [16].

Schistosomiasis is distributed across the islands of the Philippines. Out of the 81 provinces in the country, 28 are endemic and the majority of them are in the island groups of Visayas and Mindanao (Figure 2A). These provinces have been validated by local government-led snail surveys and other studies, showing the presence of infected snail intermediate host colonies, represented by *Oncomelania hupensis quadrasi*, (Figure 2A) within their communities [6,7,8,9,10,11,12]. Many of these provinces experience year-round rainfall, which is the single most important environmental factor in the distribution of snail habitats and schistosomiasis [13].

It is estimated that there are 12.4 million Filipinos living in endemic communities at-risk of exposure to schistosome, with 2.7 million identified to be directly exposed to contaminated waters [14]. Based on the 2014–2018 focal surveys encompassing most of the endemic barangays, a national prevalence of 4.8% was established. Barangays (villages) are the smallest administrative units that make up cities and municipalities. However, the burden at the province-level (Figure 2B) is heterogenous with widespread infection and distribution in Visayas, particularly in the eastern region. In addition to humans, domestic animals, particularly bovines, are persistently infected in these areas.

The distribution and prevalence of schistosomiasis are influenced by many environmental, socioeconomic, and biological factors. The disease is a significant public health and economic burden that necessitates integrated, multisectoral efforts that address its complex epidemiology (Figure 1).

## 3. Economic Impact

Schistosomiasis has a significant economic impact with its public health burden and associated loss of productivity. Disability-adjusted life year (DALY), which estimates disease burden in terms of healthy years lost due to premature mortality or disability caused by a disease in a population, can be used as a measure of lost productivity, albeit controversial [17,18]. In 2021, the worldwide DALY for schistosomiasis was 1.863 million [19].

In the Philippines, a DALY of 22,300 in 2021 was estimated for schistosomiasis [19]. In the endemic communities of Leyte, sickness due to schistosomiasis was estimated to result in the loss of 45.4 productive days per infected person per year. The loss is reduced to 4 days after treatment [20]. Another study estimated the clinical cost burden and economic losses of 1415 hospitalized cases in 2013 to be PhP 8,489,524.39 (Philippine peso) (USD 200,006.70) and PhP 13,019,363.75 (USD 306,726.25), respectively. Moreover, they determined that the average cost of schistosomiasis treatment is equivalent to 22 to 44 days-worth of wages of an average minimum wage earner, reaching catastrophic spending (≥40% of half annual income) if the poorest quintiles are affected [21].

Infection of carabao may also impact agricultural productivity, although some evidence of self-cure resulting in reduced morbidity is observed among bovines [22]. More debilitating in animal health and economically are co-infections with other parasites, such as the liver fluke *Fasciola gigantica,* which is observed to co-infect with schistosomiasis in 60% of bovines in endemic barangays in Norther Samar [23]. The liver fluke causes fasciolosis, which is associated with reduced growth rate and scouring in calves, and progresses to liver damage in chronic infections, resulting in early death and financial losses [24,25,26].

## 4. Prevalence and Spatial Distribution

Schistosomiasis is distributed across the three major island groups of the Philippines, with widespread distribution in Visayas and more focal distribution in Luzon and Mindanao [27]. The number of endemic areas in the country increased in the past decades with the addition of Cagayan Valley in 2002 and Negros Occidental in 2005 [28]; new endemic villages were discovered in Cagayan Valley as late as 2018 [7].

Historically, national prevalence surveys had been conducted covering individuals in endemic and non-endemic provinces through microscopic examination of 1 to 2 stool samples for the presence of parasite eggs using the Kato-Katz (KK), a stool-based microscopic technique. The latest national prevalence survey for schistosomiasis (Supplementary Table S1) conducted in 2013 to 2015 conducted one-stool KK on school-aged children (5 to 16 years old), establishing a national prevalence of 0.8 to 1.0% (1.7% in endemic areas). More than a quarter of the affected children were identified to be at least moderately infected [29]. The more inclusive 2005 to 2008 survey (individuals 2 years and above), supplemented by a cross-sectional study of 50 villages in Western Samar, identified the national burden of the disease to be 2.00% based on two-stool KK [27,30,31,32].

Both surveys continue the trend of decreasing national prevalence that started and peaked in 1985 at 10.4%. However, such extensive yet shallow surveys do not capture the highly focal nature of the disease wherein endemic barangays exhibit highly clustered and spatially variable distribution even within the same area [27,33].

To better define the distribution of schistosomiasis in their areas, provincial governments moved to conducting focal surveys in their known endemic barangays generating barangay-level prevalence data based on one-stool KK [16]. Overall prevalence of the village-level surveys conducted between 2014 and 2018, covering individuals aged 5 to 65 years old, was 4.8%. More pertinent is the classification of the 1611 endemic barangays based on levels of endemicity or prevalence, namely >5% as high prevalence, 1–5% as moderate endemicity, and <1% as low prevalence (Supplementary Table S1). Based on the compiled data from 2015 to 2020 surveys, 28.1% of the endemic barangays have high prevalence, 23.7% have moderate prevalence, 11.7% have low endemicity, and 15.0% have zero prevalence. More than half of the known endemic barangays are in the Eastern Visayas region encompassing the islands of Samar and Leyte (Figure 2B). Provinces on these islands have an overall high endemicity. However, the top provinces with highest prevalence are located elsewhere: Oriental Mindoro in Luzon with a prevalence of 9.5%, Lanao del Norte in Mindanao at 8.2%, and Cagayan Valley at 6.6% (Figure 2B). Other provinces in Mindanao have moderate to low endemicity, although many endemic barangays in the region lack focal survey data [16]. Previous national surveys noted the difficulty of conducting survey in areas in Mindanao, particularly Maguindanao and Zamboanga City, due to accessibility and safety given the conflicts in region [29,30].

The traditional test of KK has been shown to have limited sensitivity, particularly in low-intensity infections, resulting in potentially underestimated prevalence estimates [33]. Other studies have been conducted on endemic villages (Supplementary Table S2) utilizing different diagnostics in addition to KK, namely molecular polymerase chain reaction (PCR)-based tests, immunological tests (enzyme-linked immunosorbent assay (ELISA), the point-of-care circulating cathodic antigen (POC-CCA) test, and ultrasound, revealing even higher burden of the disease. Reviews of current and upcoming schistosomiasis diagnostics are available in Weerakoon et al. and Rivera et al. [34,35].

The majority of these studies were conducted in the Eastern Visayas region where most of the endemic barangays are located. Using two- to three-stool KK, surveys in the region detected a high prevalence (18.5–35.3%) in endemic villages [36,37,38,39,40,41]. In quantitative PCR (qPCR) testing of stool samples, these estimates reach up to 90.2% in six barangays in Northern Samar [39] and 92.3% in a village in Western Samar [23]. Moreover, the highly sensitive digital droplet PCR (ddPCR) was successfully used to detect cell-free parasite DNA in serum, urine, and saliva samples of participants in 18 barangays in Northern Samar [40], with the prevalence highest using serum samples at 67.2% and comparable with fecal ddPCR at 74.5% [40,42]. Elsewhere, surveys in Sorsogon in Luzon and Surigao del Norte correspondingly showed higher positivity rates of molecular tests than KK [43,44].

Serum and urine samples have also been used to detect antibodies against various schistosome antigens, but are problematic with significant cross-reactivity and low specificity due to co-infection with other parasites such as helminths and the persistence of anti-schistosome antibodies despite successful treatment [45]. In Eastern and Western Samar, serum-indirect ELISA using crude parasite extract detected comparably high estimates between endemic and non-endemic municipalities where there was significant co-infection of soil-transmitted helminths (STHs) [44]. Antigen detection tests such as POC-CCA should be more appropriate in detecting current infections, but have been shown to have poor sensitivity (17.1–24.1%), particularly in low prevalence settings, and poor specificity in both *S. mansoni* testing in Brazil and *S. mekongi* detection in Lao PDR [46,47,48,49]. For *S. japonicum*, POC-CCA testing of urine samples in Northern Samar recorded a prevalence (12.4%), with only about half of the KK estimate (26.2%) showing poor sensitivity [50].

Ultrasound has also been used in Leyte and Sorsogon to detect schistosomiasis-associated hepatosplenic morbidities, such as liver fibrosis, splenomegaly, and hepatomegaly, generating a prevalence of 12.3–46.67% [41,43]. However, results from ultrasound are only suggestive and more applicable in identifying chronic schistosomiasis [41].

While many areas remain endemic, as early as 1995, there were three provinces already deemed to be near elimination, namely Zamboanga del Norte, Bohol, and Davao Oriental [51]. These provinces maintained 0 to less than 1% prevalence in national and focal surveys (Supplementary Table S1), although one village in Zamboanga del Norte was identified to have moderate prevalence, while no focal survey has been accomplished in Bohol [29,30,31]. Nonetheless, these sustained low incidences of the disease across surveys are indicative of significantly reduced transmission of the parasite. In a transmission model, endemic villages in northeastern Bohol were predicted to sustain near-elimination prevalence in light of effective selective treatment and snail control (weed clearance, mollusciciding) that began in 1981 and is continuing as of 2008 [52]. However, current schistosomiasis control activities of the Bohol provincial government shown in their website no longer include mollusciciding [53]. Another province candidate for near elimination is Davao del Sur based on the focal surveys in 2018, which identified zero cases in all of the 14 endemic barangays (Supplementary Table S1) [16]. Interestingly, provinces showing near-elimination prevalence had been the initial targets for 100% coverage of case finding and treatment that started in 1988 [13]. Naturally, sustained and focused implementation of control efforts is crucial to the elimination of schistosomiasis, which may be difficult to execute in areas with extensive distribution such as Eastern Samar. Still, a number of villages (15.9% of known endemic barangays) in the region reached zero prevalence based on the focal surveys [16].

The focal surveys of known endemic areas improved the resolution of the disease distribution to village level, but what is also important is the monitoring of non-endemic areas for emergence of the disease. The past nation-wide surveys detected at least one case in non-endemic areas: the National Capital Region (NCR), the city of Iloilo, and the provinces of Ilocos Norte, Biliran, Dinagat Islands, Basilan, and Tawi-tawi [29,30,31]. These cases may be the result of the migration of infected individuals from endemic to non-endemic areas as determined in the two cases detected in NCR that came from Cagayan [30]; the origin of the other cases have not been investigated. Especially important for monitoring are non-endemic areas where colonies of snail hosts are also found, such as Ilocos Norte, Nueva Vizcaya, Tarlac, Quezon and Palawan in Luzon, and Aklan and Cebu in Visayas [54]. Contamination of these snail habitats with parasite eggs from feces of infected mammalian hosts could potentially result in the establishment of new endemic foci.

### 4.1. Environmental Factors to Spatial Distribution

The focal distribution of schistosomiasis is primarily determined by the distribution of the snail intermediate host, reflecting the specific environmental requirements of both the snail and the parasite. *O. h. quadrasi* has a specific ecological niche generally characterized as shallow (i.e., depth less than 20 cm), slow-moving freshwater wetlands on a loamy soil with thick vegetation and minimal disturbance [11,55,56]. The snails are amphibious and require constant moisture, with snail hatchlings aquatic for 1 to 2 weeks. Vegetation prevents evaporation of water and provides cool shade, working in favor of their aversion to high temperature and intense light, which regulates much of their behavior, including copulation and hatching as well as providing objects for climbing, which is a behavior linked to sexual activity. Moreover, plants and the loam soil are the sources of organic material that sustains their diet as dirt-feeders [55,57,58,59].

Natural snail habitats in the Philippines take the form of flood-plain forests and swamps. Many of these snail environments are suitable for agriculture and many had been developed for farming, diminishing these natural abodes. However, the snails have proven themselves adaptable, maintaining colonies in agricultural developments such as irrigation canals and abandoned or primitively farmed rice fields with minimal human disruptions, and expanding to road ditches [55,58].

Based on an extensive snail mapping conducted by the Philippine Department of Health (DOH) from 2014 to 2019 with data available for all endemic provinces except Oriental Mindoro and covering a total of 3132 snail sites, 34.6% were found to have the snail intermediate host, and among them, 27.3% were identified to be contaminated with *S. japonicum* [6]. Except for Maguindanao, all of the surveyed provinces had at least one infected snail site, with the most numerous found in Bukidnon (67 infected snail sites) and Northern Samar (58). In Mindanao, the provinces with the most identified infected snail sites were Agusan del Sur and Surigao del Norte (19 and 14 infected snail sites, respectively) [6].

Pesigan and others identified in 1958 that the most extensive natural habitats of the intermediate host that remain untouched lie in the lowland forests in Mindanao flooded by nearby rivers. The largest of which they discovered is the forested Manat River swamp in Davao de Oro, where they found *O. h. quadrasi* snails on the forest floor and along a trail crossing near the swamp center [55]. Another survey in the Davao region identified snail colonies in a forest swamp and another in a borrowed ditch near a forest [59]. The Manat River is one of the tributaries of the Agusan River drainage basin that runs along the endemic provinces of Davao de Oro, Agusan del Sur, Surigao del Sur, and Agusan del Norte. The longer Mindanao River is located on the eastern part of Mindanao with its tributaries spanning the endemic regions of Northern Mindanao and Soccsksargen. These river systems supply the water in these low-lying wetlands such as swamps, marshes, and floodplains where snail colonies are established.

Other freshwater sources, such as springs forming swamps, streams and creeks, are also potential water sources of snail habitats as identified in Sorsogon, Samar, and Leyte [9,11,55,59]. In Eastern Visayas, snail surveys identified flowing creeks and streams to be common snail habitats in Samar provinces and swamps in Leyte [9,11]. Snail colonies in Bohol include creek, palawan, and boggy, with Ipil River being the main freshwater in the area [60]. Lake Mainit in the Caraga region and Lake Naujan in Oriental Mindoro sustain schistosome-infected snails and are known focal points of transmission in these areas [12,61]. Similarly, in China, the country’s two largest lakes, Poyang Lake and Dongting Lake, are major areas of transmission, contributing to 95% of snail habitats along with marshlands [3,62].

Areas surrounding Lake Mainit encompassing endemic villages in Agusan del Norte and Surigao del Norte have been transformed to agricultural lands determined to be inhabited by schistosome-infected snails with a prevalence of 21.45% [12]. Unsurprisingly, in a review of *O. h quadrasi* habitats in the Philippines, agricultural lands have been determined to be the most common territories occupied by snail populations [54]. In Compostela Valley, infected snails were found in stagnant rice fields [9], while snail habitats in the mostly dry Cagayan include irrigation canals, swamps, streams, and ponds shaded with vegetation [7,8].

#### 4.1.1. Macro-Environment

Rainfall, or the lack thereof, is the single most important environmental factor in the distribution of snail habitats and schistosomiasis. Minimal environmental disturbance and topography, specifically low elevation, are other factors that define the suitability of a habitat for *O. h. quadrasi*. Most of these habitats, natural and man-made, are ultimately generated and maintained by rainfalls [55]. Rainfall also positively regulates much of the behavior of the snails and the parasites, such as snail egg laying and release of cercariae [55,58]. Aside from providing moisture, heavy precipitation may result in a sweeping flood which provides a mechanism that can expand and modify snail distribution, transporting parasite-infested snails to new foci. In the wake of the super typhoon Haiyan (Yolanda) in Leyte in 2013, emergence of and disappearance of snail habitats in endemic villages, and establishment of new schistosomiasis foci were identified [58].

The importance of rainfall in schistosomiasis distribution is supported by the observation that all of the original endemic provinces are limited to areas with climate types with no dry season: type II in, which rainfall peaks from November to January, and type IV, in which there is even distribution of rainfall throughout the year [13,51]. However, the more recent discovery of endemic foci in Gonzaga, Cagayan, which generally had a defined dry season from December to February (type III) with summer temperatures reaching a maximum temperature of 40 °C [7,28], indicates some form of tolerance to high heat by the intermediate host. Indeed, the snails can aestivate to survive periods of dryness for up to 3 months [58].

Moreover, in an ecological niche model investigating the contribution of bioclimatic variables to snail site distribution in the Philippines, the authors determined that the most influential factors relate to an area’s dry season, the most important being mean temperature of driest quarter followed by factors relating to precipitation in dry parts of the year [54].

In a national predictive risk map for schistosomiasis, the effects of some environmental factors related to snail distribution to schistosomiasis prevalence were found to vary in different areas of the country (i.e., non-stationary spatial process) [27]. Rainfall was determined to positively correlate with prevalence in Visayas, but the opposite is observed in Mindanao, where the effect of topography has been suggested to explain the establishment of snail colonies in the area. In terms of increasing the normalized difference vegetation index (NDVI), which is a measure of vegetation greenness, prevalence in Luzon increases, but decreases in Visayas, which may reflect difference in vegetation between the island groups. Distance to perineal water bodies (DPWB) is inversely associated with prevalence in Luzon and Visayas [27]. Indeed, proximity to a water source has been determined to be a reliable indicator of schistosomiasis [63].

These predictive models are useful at identifying potential areas where snail colonies exist and are at risk for schistosomiasis transmission. Using the ecological niche model, highly suitable climatic conditions are present in the non-endemic provinces of Quezon, specifically Polillo Island, Camarines Norte, Camarines Sur, and Albay [54]. The schistosomiasis risk map published in 2014 predicted potential high prevalence areas in Luzon, albeit with low predictive ability (area under the curve (AUC) 70%), the coastal and central parts of Isabela, the northern border between Quirino and Nueva Vizcaya, the Cordillera Administrative Region, and the CALABARZON Region between Laguna and Batangas, a very small area in central Palawan and the province of Albay [27].

These areas, along with known non-endemic areas where snail colonies are observed (e.g., Ilocos Norte, Nueva Vizcaya, Tarlac, Quezon, and Palawan in Luzon, and Aklan and Cebu in Visayas) [54] are potential endemic foci. Typhoons worsened with climate change in the country and the more expansive flooding may transport infected snails to these vulnerable areas and possibly lead to the emergence of new endemic areas as already shown in the aftermath of typhoon Haiyan [58,64]. Moreover, the increase in temperature associated with climate change is forecasted to expand schistosomiasis to non-endemic areas as identified in China [65].

#### 4.1.2. Micro-Environment

Pesigan and others observed that many potential habitats do not carry the intermediate host and hypothesized the role of physicochemical factors but found no association [55].

A scoping review (2021) of micro-environmental factors associated with *O. hupensis* identified abiotic and biotic factors with impacts on snail survival, reproduction, and density [66]. They reviewed that snail habitats exist in an optimal range of various soil and water factors, i.e., a flooding time of 2–7 months, pH of 5.5–7.9, soil temperature of 15–30 °C, and humidity of 20–80% [66]. Surveys of snail habitats in Cagayan and Bohol identified positive effects of factors indicative of high soil nutrient content (high soil organic matter, calcium oxide (CaO)) [7,60], while a Chinese survey was inconclusive about the effect of soil fertility indexes (total nitrogen, phosphorus, and potassium) [66].

The biotic characteristics of these potential habitats, such as gastropod fauna, soil microorganisms, vegetation, and snail predators, also influence snail distribution [55,66]. The presence of competing gastropods, predators, vegetation, and plant and soil microorganisms with molluscicide properties may restrict the existence of snail colonies in an otherwise viable habitat [66].

### 4.2. Snail-Parasite Geographic Strains

With the limited mobility of snails and the geographic isolation among endemic provinces, the low-to-zero gene flow between snail and parasite populations resulted in genetic variation with implications for parasite interaction and disease transmission. The species *O. hupensis* has been highly adaptable, establishing colonies in new territories as it spread from mainland China to Taiwan, then to Japan and the Philippines and Indonesia [67,68]. This adaptive radiation has been ordered to eight or nine subspecies characterized not only by geographic location, but also by morphological, genetic, and biomolecular differences [67,68]. The Philippine subspecies *O. h. quadrasi* has been shown to significantly differ from the other subspecies based on genetic and biochemical markers and has been proposed to be elevated as a separate species as early as 1988 [69,70,71,72].

Similarly, geographically isolated *O. h. quadrasi* populations have been shown to differ genetically, although to a lesser extent. An earlier study (1994) using restricted fragment length polymorphism (RFLP) analysis noted negligible genetic variation between Philippine snail populations from the islands of Luzon, Mindoro, and Leyte [71]. Sub-structuring of the snail species in the Philippines based on geographic location of six snail populations in the provinces of Leyte, Oriental Mindoro, and Cagayan was revealed using a 12S ribosomal RNA sequence [70]. The study noted significant genetic variation even among populations within the provinces of Cagayan and Oriental Mindoro. Based on a 342 base pair fragment of the 16S ribosomal gene, phylogenetic and network analysis grouped populations from Leyte, Oriental Mindoro, and Cagayan, along with Davao, Davao del Sur, and Sorsogon. A separate haplotype comprises the Bohol snail population while two haplotypes were determined for Negros Occidental isolates [73]. Using ITS markers, Surigao del Norte and Lanao del Norte isolates were determined to group together, while Leyte, Agusan del Sur, Compostela Valley, and Sorsogon snails form another [58]. Use of additional markers may be needed to paint a clearer picture of the differentiation of snail populations within the country, but the already identified genetic variations reflect the different environments and conditions of snail habitats, such as the intense molluscicide practice specifically implemented in Bohol, and the relatively new emergence of schistosome-infected snail populations in Cagayan and Negros Occidental [28,58,73,74].

Similarly, with their close evolution to their specific *O. hupensis* subspecies, *S. japonicum* populations across Asia have been shown to exhibit variations based on genomic and mitochondrial markers [75,76,77,78,79]. Such variations may reflect as parasite strains with differential virulence to different snail populations as in the Philippine and Chinese strains of the parasite, which have significant phenotypic differences in fecundity, pathology, drug sensitivity, and immunology contributed by a copy number variation in associated genes [80]. Using microsatellite and allozyme markers, genetic variations have also been observed among schistosome populations in the endemic provinces of the Philippines [81,82] and in China using microsatellite markers [83]. The Taiwan snail subspecies *O. h. formosa* was found to be resistant to the Philippines schistosome isolates, while *O. h. quadrasi* have low susceptibility to the Formosan strain of *S. japonicum* [84,85]. In the Philippines, schistosomes in Mindoro and Davao del Norte showed good infectivity to snails in both provinces, but mild to no susceptibility for snails in Leyte, resulting in sluggish cercariae [86]. Parasites isolated in Leyte can highly infect Bohol snails but not Mindanao snails, while Bohol schistosomes are less infectious to snails from Leyte and Mindanao [87].

However, comparative phylogenetics of snail hosts and parasites from China, Japan, and the Philippines showing unlikely long-term co-evolution of the parasitic relationship signified that the existence of resistant and vulnerable snail isolates to different parasite strains is potentially transient [88]. This means that *S. japonicum* strains are not confined to a particular *O. hupensis* subspecies or population, and there is a greater capacity for the parasite to spread into new areas through host switching [88]. Hence, surveillance should also include non-endemic areas known and predicted to support snail colonies to restrict the expansion of endemic areas, which may be exacerbated by climate change and increased population movement [58,65,89,90].

## 5. Schistosomiasis Transmission

Transmission is primarily driven by snails whose habitats are usually maintained by rainfalls. In China, there are distinct transmission seasons with peaks coinciding with the height of annual flooding from April to June/July and when water begins to subside from September/October to November [62]. Given the absence of a true dry season in many endemic areas in the Philippines, infectious cercariae are present throughout the year [3]. Exposure to infected areas persists due to year-round agricultural activities, including two to three rice harvests annually; exposure can also be through domestic chores (washing in infected streams) and recreation (swimming) [3,12,59].

Another important consideration for *S. japonicum* is zoonotic transmission. The relative contribution of human and animal hosts can be measured in terms of transmission index, which quantifies the proportion of viable parasite eggs released by mammalian hosts to the environment. The first study (1958) about the transmission of schistosomiasis in the country accounted for a majority of transmission in Palo, Leyte to be from humans at a 75% transmission index; the remaining quarter was contributed by animal hosts [91]. However, this result was prior to the recognition of bovines as major reservoir hosts, which we will discuss more in the next section.

### 5.1. Animal Transmission

The first report of animal schistosomiasis in the islands of the Philippines was in 1941 by Tubangui and Pasco in dogs and pigs [92]. Carabaos (*Bubalis bubalus carabanesis*), the native ‘swamp type’ water buffaloes in the Philippines, were additionally identified to harbor the parasite in Mindoro [93], then natural infections in rats, cattle, and goats in Leyte was reported in 1958 [91]. Currently, there are over 40 wild and domestic animal species that can be infected with *S. japonicum,* with domestic animals mainly considered to be epidemiologically important due to their close contact to humans [62]. Animal infection significantly contributes to transmission through environmental contamination of viable parasite eggs in the stool of infected animals, maintaining the parasite life cycle even in the absence of human infections.

Dogs were initially deemed the primary animal host for schistosomiasis in the Philippines due to early studies, including a study in fifty barangays in Samar province, showing relatively high prevalence in dogs [94]. While dogs were considered to be uncommon in endemic villages in China until recently [62], they are familiar in Filipino communities in households mainly as house guards and on the streets as strays called askal (street dog) or aspin (Filipino dog) [95,96]. Association between *S. japonicum* infection in dogs and humans had been demonstrated in various studies in Western Samar [94,97,98,99]. In a population genetics study, high frequency of parasite gene flow was particularly noted between dogs and humans, which is suggestive of a significant *S. japonicum* transmission between the two hosts [98]. However, dogs practice coprophagy and human open defecation is commonplace in these areas, muddying such interpretations.

The significance of carabao in schistosomiasis transmission in the Philippines had previously been downplayed. This was in contrast with findings in China that water buffalo are major reservoir hosts, releasing the highest number of eggs in the environment of any animal host, and accounting for 75% of transmission to humans based on mathematical modeling [100,101]. A different transmission dynamic of infection in Western Samar was purported in a 2003 to 2004 study in 50 villages, with dogs suggested as the major animal reservoir host due to a high prevalence of *S. japonicum* (19.18%), while bovines were considered unimportant based on very low prevalence (3.21%) in that study [94,97]. However, with the development of more sensitive molecular [102] and copro-parasitological tests [103], a much higher prevalence was later detected among carabao and cattle in the islands of Leyte and Samar using qPCR (51.5–90.9%) and the formalin-ethyl acetate sedimentation (FEA-SD) (55.2–93.2%), identifying these animals as major reservoir hosts and drivers of transmission in the Philippines [23,36,104].

Similarly, serological testing of carabaos in Cagayan, Negros Occidental, Northern Samar, and Bohol indicated high incidences of *S. japonicum* infection (10.7–69.4%) [105,106]. Moreover, Jiz and colleagues detected an infection rate of 97% among carabao introduced to a rice farming village in Leyte in less than a year, further establishing their importance as reservoir hosts [107].

Infected bovines have been identified to release tens to hundreds of thousands of parasite eggs with their stool per individual per day, measured as the bovine contamination index (BCI) based on mean egg per gram of stool (EPG) and prevalence (Table 1). In total, bovine schistosomiasis surveys in endemic villages in Eastern Visayas identify the egg environmental contamination of millions per day.

Animal surveys conducted in 1980s and earlier identified that wild rats (*Rattus rattus mindanensis*) have the highest prevalence (81.9%) among animals in endemic villages [91,108,109,110]. High prevalence in rodents was highly correlated with proximity to snail habits, with decreasing prevalence the farther away from snail colonies [111]. It was argued that rats do not largely contribute to the spread of disease from snail colonies to human communities, and that despite the high prevalence, rodents have minimal epidemiological significance and are considered as non-permissive hosts due to their biology that limits the generation of viable parasite eggs [111,112,113]. However, transmission models found association between human infection and rat infection, highlighting their significance as schistosomiasis sentinel, serving as indicators of nearby infected snail intermediate host colonies [99,114].

Other relevant important animal hosts are pigs and goats, which are more common livestock in Philippine farms than bovines, and they are mostly situated in smallholdings [115]. They are deemed epidemiologically unimportant in China as pigs are short-lived and mostly in pens, limiting contamination of their waste to the environment, while goats and sheep have low fecal output and only stay in endemic areas for a limited time as they are sold at an early age [62]. Such may not be the case in the Philippines, as pigs are not strictly limited in pens and goats are mainstays in farms. In an endemic community in Alegria, Surigao del Norte, the presence of a pig farm with unregulated waste disposal was associated with a nearby snail site that had a positive snail prevalence of 14.71% [10].

**Table 1:** Bovine prevalence and infection intensity in the Philippines.

Province	Municipality/ City	Year	Animal	Diagnostic	No. Animal	No. Positive	% Prevalence	Mean EPG	BCI per Bovine	Total BCI	Reference
Leyte	Jaro	2008	Water buffalo	qPCR	66	34	51.52%	2.05	51,250	1,742,500	[104]
Northern Samar	Palapag	2012	Water buffalo	qPCR	105	84	80.00%	14.1	352,500	29,610,000	[23]
				FEA-SD	105	58	55.24%	5.5	137,500	7,975,000	
			Cattle	qPCR	48	42	87.50%	15.4	385,000	16,170,000	
				FEA-SD	48	37	77.08%	11.4	285,000	10,545,000	
Western Samar	San Jorge	2010	Water buffalo	FEA-SD	44	41	93.18%	1.67	41,750	1,711,750	[36]
				qPCR	44	40	90.91%	6.14	153,500	6,140,000	
				KK	44	11	25.00%	4.69	117,250	1,289,750	
South Cotabato	Koronadal City	2018	Water buffalo	FEA-SD	38	23	60.53%	3.36	84,000	1,932,000	[116]
			Cattle	FEA-SD	70	34	48.57%	1.97	49,250	1,674,500	
Agusan del Norte	Jabonga	2020	Bovine	Modified FEA-SD	6	3	50.00%	1.4	35,000	105,000	[12]
Surigao del Norte	Alegria	2020	Bovine	Modified FEA-SD	12	2	16.67%	0.8	20,000	40,000	

EPG—eggs per gram of faeces; BCI—bovine contamination index; qPCR—real-time/quantitative PCR; FEA-SD—formalin ethyl acetate sedimentation technique; and KK—Kato-Katz.

### 5.2. Human Transmission

Pesigan’s seminal study (1958) in schistosomiasis transmission accounted the majority of transmission in Palo, Leyte to be from humans [91]. Humans far outnumber the population of domestic and farm animals in endemic communities. Based on the computed transmission index, about 3/4 of parasites in the endemic village were shown to be originated by humans, with the age group 10 to 14 years old contributing the most at 59.9%. The same age group was found to be the most affected by the disease [91]. There has been little research since on humans as potential drivers of infection.

#### 5.2.1. Biological Factors to Susceptibility

The occurrence of *S. japonicum* has been shown to differ with age and sex. In general, males and individuals aged ≥20 years had significantly higher prevalence than females and children aged <5 years [27]. In general, there is an increase in prevalence in young children to adolescence (<20 years old), reaching a peak in the early to mid 20s, then generally stabilizing thereafter with additional peaks at around 40 years, as reflected in the 2005 to 2008 national prevalence survey [30,31]. A similar age–prevalence distribution is also observed in endemic populations in China [117].

Different biological processes have been implicated for the observed higher susceptibility to infection and reinfection of the adolescent cohort, such as age-dependent immune response and post-pubertal hormonal influences [117,118,119]. Resistance to reinfection has been shown to be mediated by repeated release of immunogens induced by repeated exposure to worm death either naturally or via the action of praziquantel, taken as treatment or by mass drug administration (MDA) against schistosomiasis. Over time these immunogens can induce protective responses by reacting with schistosomula antigen or decrease in fecundity [118]. Such age-dependent acquired immunity had been demonstrated to involve both humoral responses mainly involving IgE and cellular mechanisms (Th1-type involving IFN-γ and IL-2 and Th2 type involving IL-4, IL-5, and IL-10) [117]. IgE against adult worm was found to increase with age in a Philippine population. In *S. japonicum*, the 22.6-kDa worm antigen was identified as a major target of the human IgE response [120], while the recognition of its ortholog in *S. mansoni* (Sm22.6) has been correlated with a low reinfection rate post treatment [120,121].

Puberty has also been suggested to mediate the resistance observed in older individuals. Adrenarche marks the early stage of sexual maturation during which androgens are released by the adrenal glands, contributing to some of the changes observed during puberty. These androgens peak at around 20 years of age. One of them is dehydroepiandrosterone sulfate (DHEA-S), which has been associated with a lower intensity of baseline *S. japonicum* infection and reinfection in a cross-sectional and longitudinal study [119]. DHEA-S has been shown to reduce worm burden in mouse models and kill larval and adult parasites in culture. It was suggested that DHEAS reduced infection intensity by downregulating pro-inflammatory immune responses, thereby alleviating undernutrition and anemia among the infected individuals [122].

In terms of sex, males are found to be at a higher risk of infection than females, with prevalence in males being 2–4 times higher than females in the country [30,31]. Such sex differences have been noted to be present only after childhood [91], likely due to most agricultural activities being performed by males, which is a risk factor for infection due to higher water contact, and therefore presents a higher chance of exposure to infectious cercariae, while children are more likely to become infected by recreational use. Moreover, the relationship between *S. japonicum* infection and malnutrition is stronger in males, indicative of gender-dependent response to schistosomiasis resulting in higher morbidity in males [123]. In *S. haematobium* infections, males were shown to have significantly increased production of proinflammatory cytokines TNF-α and IFN-γ and decreased production of TGF-β and IL-10, indicating that higher morbidity may be due to a stronger inflammation response in males [123].

#### 5.2.2. Socioeconomic

Socioeconomic factors and individual behavior have been proposed to account for the smaller-scale focal distribution of schistosomiasis [27]. Indeed, low socioeconomic status and poverty have been recognized to be highly predictive of infection status for both STHs and schistosomiasis, as demonstrated in Northern Samar, which is one of the poorest regions the country [63].

In the Philippines, poverty itself is shown to be spatially dependent, with physical geography found to explain a significant percentage (40.2–54.2%) of province-level variation in human development indicators, including life expectancy, mean years of schooling, per capita income, poverty incidence, and human development index (HDI) [124]. The highly diverse topography and climate patterns, particularly relating to rainfall and typhoon, across the Philippine islands have economic and health implications, such as unequal access to market centers and health services, varying land productivity, vector distribution of various NTDs, and different susceptibility to natural disasters such as typhoon [125]. Similar to schistosomiasis, poverty is more prevalent in the Visayas and Mindanao. Eastern Visayas is vulnerable to typhoons, such as typhoon Yolanda (Haiyan) in 2013, plunging communities into further poverty [126]. Mindanao is the farthest region from the main economic center of Metro Manila and is ceaselessly stricken by armed conflicts [125]. There is a significant overlap between regions with schistosomiasis and regions with high poverty and subsistence poverty incidence (Table 2) [127]. Literacy and HDI (Table 2) are higher in general in Luzon and in Western and Central Visayas where schistosomiasis distribution is present only in small localized areas [128,129].

Low socioeconomic status has been associated with increased risk for infection and greater disease transmission [63,123,130]. Poor nutrition among children has been shown to increase susceptibility to schistosomiasis [37,123], while agriculture and lack of hygiene and sanitation infrastructure in these low socioeconomic villages contribute to the persistence and spread of schistosome-infected snail habitats and increased exposure to contaminated waters [27,131].

**Table 2:** Socioeconomic statistics of regions of the Philippines associated with schistosomiasis.

Region	No. Schistosomiasis Endemic Barangays	Economic and Human Development Indicators				Agriculture in the Philippines								Household Cooking Water				Household Sanitation					
		Poverty Incidence Among Pop’n (%) (2023)	Subsistence Incidence Among Pop’n (%) (2023)	Literacy (2020)	HDI (2012)	Poverty Incidence Among Farmers (%) (2021)	Rice Farms Rain-Fed (ha) (2022)	% Total Rain Fed (2022)	Rice Farms Irrigated (ha) (2022)	% Total Irrigated (2022)	Farm Mechanization Level (hp/ha) (2022)	Carabao Inventory (Thousand Heads) (2023p)	% Employed In Agriculture (2021)	Community Water System	Piped, protected Well/Spring	Unprotected	Commercial	Sewer/Septic	Improved Latrine	Pit Latrine	Toilet to Open/Unknown	No Facility	% Barangay ZOD Certified (2022)
**PHILIPPINES**	1611	22.36	8.68	97%	0.64	30.00	1,463,680.17	100.00%	3,340,910.40	100.00%	2.68	2740	100.00%	63.91%	23.98%	3.59%	7.27%	79.71%	2.37%	10.13%	2.69%	3.31%	32.32%
**National Capital Region (NCR)**	--	5.06	0.88	99%	0.83	--	--	--	--	--	--	--	5.15%	90.79%	1.25%	0.37%	6.30%	91.61%	0.33%	1.29%	1.62%	0.14%	37.02%
**Cordillera Administrative Region (CAR)**	--	13.88	4.93	96%	0.54	15.60	18,084.20	1.24%	79,483.83	2.38%	2.35	80.36	0.10%	44.32%	39.03%	9.10%	5.07%	76.84%	12.74%	7.74%	1.48%	0.96%	28.89%
**Region I (Ilocos Region)**	--	18.67	7.31	99%	0.66	17.20	123,749.00	8.45%	283,659.35	8.49%	3.09	143.63	1.95%	42.42%	49.43%	1.22%	6.49%	82.41%	2.25%	11.28%	1.58%	0.85%	84.30%
**Region II (Cagayan Valley)**	5	16.15	4.65	98%	0.58	18.80	72,125.86	4.93%	549,932.44	16.46%	3.51	208.71	0.84%	33.54%	59.41%	2.70%	4.07%	76.44%	3.93%	15.35%	2.00%	1.32%	15.88%
**Region III (Central Luzon)**	--	15.69	4.37	98.32%	0.64	17.40	29,195.65	1.99%	675,882.81	20.23%	2.62	250.64	9.30%	67.18%	20.50%	0.75%	11.02%	87.82%	1.26%	6.79%	1.28%	0.97%	19.66%
**Region IV-A (CALABARZON)**	--	13.48	3.93	98.47%	0.70	20.70	27,202.59	1.86%	78,409.11	2.35%	3.36	183.37	6.18%	76.89%	11.31%	1.16%	9.83%	81.05%	1.57%	13.12%	1.54%	1.29%	6.00%
**MIMAROPA REGION**	33	26.85	9.99	95.56%	0.56	29.50	100,678.76	6.88%	213,986.53	6.41%	2.22	152.81	1.62%	47.03%	38.72%	8.58%	4.07%	70.43%	4.09%	12.53%	2.73%	9.34%	7.81%
**Region V (Bicol Region)**	15	32.87	12.78	97.45%	0.51	34.60	120,007.68	8.20%	228,840.33	6.85%	2.98	324.22	1.58%	56.38%	32.09%	5.41%	3.99%	74.69%	3.59%	10.29%	2.09%	8.37%	27.31%
**Region VI (Western Visayas)**	3	25.98	10.08	97.69%	0.61	27.70	382,152.43	26.11%	299,147.82	8.95%	2.76	251.57	11.31%	37.27%	42.78%	5.31%	13.64%	71.83%	2.01%	16.83%	2.56%	5.66%	69.46%
**Region VII (Central Visayas)**	8	31.04	13.71	97.13%	0.61	41.20	31,811.09	2.17%	57,405.79	1.72%	3.02	218.29	5.97%	67.01%	20.72%	4.21%	7.26%	81.54%	1.42%	7.53%	1.68%	6.85%	14.42%
**Region VIII (Eastern Visayas)**	873	33.71	14.89	95.43%	0.49	41.70	125,519.63	8.58%	118,650.35	3.55%	2.41	163.03	1.57%	64.50%	25.71%	4.13%	4.66%	80.92%	2.45%	5.94%	1.93%	7.70%	43.62%
**Region IX (Zamboanga Peninsula)**	81	38.15	17	93.96%	0.51	49.10	67,236.12	4.59%	95,132.55	2.85%	1.59	142.19	5.46%	61.50%	25.35%	6.83%	4.31%	67.52%	4.50%	15.29%	6.84%	4.78%	13.29%
**Region X (Northern Mindanao)**	112	32.77	13.36	96.02%	0.53	35.40	23,190.87	1.58%	150,212.73	4.50%	2.3	124.94	14.43%	72.91%	19.10%	4.39%	2.90%	81.15%	2.70%	9.24%	2.79%	3.07%	42.09%
**Region XI (Davao Region)**	41	27.16	13.89	96.79%	0.52	24.60	14,119.5	0.96%	96,453.19	2.89%	2.59	123.31	22.30%	64.69%	21.09%	3.91%	8.72%	88.04%	2.21%	4.86%	2.16%	1.85%	7.92%
**Region XII (SOCCSKSARGEN)**	45	29.97	13.98	94.71%	0.48	35.10	72,243.14	4.94%	268,580.74	8.04%	1.72	194.52	9.66%	54.74%	36.80%	4.46%	3.08%	75.41%	4.62%	12.76%	3.33%	3.09%	28.50%
**Region XIII (Caraga)**	298	28.09	11.03	96.40%	0.51	42.50	67,967.27	4.64%	91,551.75	2.74%	1.65	66.62	2.05%	67.31%	21.37%	5.57%	4.35%	59.24%	2.51%	31.91%	2.00%	3.74%	26.93%
**Bangsamoro Autonomous Region in Muslim Mindanao (BARMM)**	28	44.79	18.07	86.42%	0.33	34.70	188,396.38	12.87%	53,581.07	1.60%	0.93	108.61	0.54%	32.65%	37.74%	19.36%	2.75%	43.39%	5.79%	15.47%	21.78%	9.91%	21.37%
**Reference**	[16]	[127]		[128]	[129]	[132]					[133]	[134]	[132]	[128]									[135]

ZOD = zero open defecation; HDI = human development index.

##### Agriculture

Agriculture remains to be an important industry in the Philippines despite its decreasing contribution to the country’s GDP. While it contributed 8.94% in the country’s GDP in 2022, the agricultural sector still employed almost a quarter of the country’s workforce [136]. Agriculture in the Philippines is highly associated with poverty, with the majority of poor households mainly reliant on the industry [137]. Poverty incidence among farmers is higher than the general population in many regions of the country (Table 2) [132]. Aside from being an indicator of poverty, the practice of agriculture itself majorly contributes to schistosomiasis distribution and transmission.

Many farm lands in endemic areas are original snail habitats, and agricultural developments additionally generated potential habitats in irrigation canals and road ditches [55]. The conversion of these wetlands to farms and the subsequent establishment of communities around these areas facilitated by irrigated farming decreased the distance between infected snail habitats and human settlements [11,55]. Comparing irrigated and rain-fed villages, a survey in Western Samar did not find significant difference in human schistosomiasis infection [32], but identified more occurrence of infected snails in streams and springs near irrigated villages [10]. Moreover, irrigation enables rice farming even during the dry season and provides snail habitats not reliant on rainfall. The region of Cagayan Valley constitutes the second largest irrigated land (Table 2) in the country where irrigation canals in endemic areas were found to sustain infected snail colonies, demonstrating that schistosomiasis can expand to mostly dry areas in the presence of artificially maintained wetlands [7,138].

Most susceptible to infection in these agricultural foci of transmission are farmers and irrigation workers, as they labor in these contaminated lands and irrigation canals, continuously exposing them to infectious cercariae. People who did not work on a rice farm had been shown to have the lower odds of becoming infected than those working full-time on rice farms [32]. Farming is a male-dominated occupation and has been implicated as a cohort for having the highest exposure, explaining the higher prevalence among men than women [139] and the persistence of high prevalence among adults despite age-related immunity to reinfection [117]. Another study, however, determined no significant difference in water exposure between males and females in Barangay Macanip, Leyte and determined that females are more likely to be less susceptible [140]. Many household chores, mostly managed by the mothers, may also involve exposure to potentially contaminated waters, such as laundry of clothes and washing of dishes in streams and lakes [131].

These freshwaters are also common wallowing places for carabao, where they may be infected; carabaos are used as draught animals in rice farms, which are another source of infection [55,91,107]. Carabaos have been established to be important in maintenance of the parasite lifecycle in China and the Philippines, sustaining large numbers of schistosome parasites per animal as reservoir hosts and releasing hundreds of thousands of worm eggs to the environment, accounting for up to 90% of total egg contamination [23,62,107]. Mechanization of agriculture in China to replace these reservoir hosts had been shown to significantly reduce prevalence in humans and bovines [141]. In the Philippines, carabaos remain common agricultural aides, with 2.7 million carabaos working in farms across the country in 2023, while mechanization level is low (Table 2) [133,134]. Many farmers show willingness to shift to mechanized farming, which, in a pilot scale implementation in an endemic municipality in Leyte, resulted in an increased profit of up to 10 times for 2000 farmers, demonstrating that the intervention will not only reduce parasite transmission, but also alleviate poverty [142].

Other farm animals may also sustain the parasites and further contribute to environmental contamination, particularly unfenced animals with uncontained waste management. Risks from farm run-off are tackled in China through fenced husbandry, minimizing both animal exposure and environmental contamination [141], which could also be implemented in the Philippines.

##### Poor Water, Sanitation, and Hygiene (WASH)

Poor WASH is another dimension of poverty associated with schistosomiasis transmission. In a review about the impact of WASH on schistosomiasis, the authors concluded that improved WASH significantly reduces the risk for schistosomiasis, but specificities are less clear [131,143].

Safe drinking water from piped water sources does not prevent exposure to infectious cercariae, as most instances of infection are from occupational and recreational activities in contaminated waters or wetlands [131]. Water systems where water flow are not regulated, and lack of water drainage systems have been suggested to generate puddles of water where snail colonies may establish [6]. Based on the 2020 Census of Population and Housing by the Philippines Statistics Authority (PSA), commercial water from refilling stations or bottled water is the most common drinking water source in most of Luzon, Western and Central Visayas, and Caraga in Mindanao, while the remaining regions most commonly utilizes tap water from community water systems for drinking [128]. Water sources for cooking may be more reflective of the main water source installed in a household wherein the majority of households either use community water systems or a protected well/spring, but a significant percentage of households (3.59%) mainly rely on (Table 2) an unprotected well/spring or rainwater. All regions in Visayas and Mindanao, CAR, MIMAROPA, and Bicol region register higher use of unprotected water sources compared to the national average. In particular, use of unprotected water for cooking is exceptionally high in BARMM, with 19.36% of households reporting the use of unprotected water sources [128].

Access to piped water for sanitation may be more relevant to schistosomiasis transmission given the preference among Filipinos and most of Asia to clean up with water following defecation [144]. The presence of piped water in toilet discourages the practice of hygienic bathing in open freshwater to clean up residual feces on the body or clothing, potentially contaminating the environment with parasite eggs [131].

Even more crucial are household sanitation facilities that contain human feces from the environment and discourage open defecation [131]. Except for BARMM, the majority of households have toilet facilities that drain in situ to sewer systems or septic tanks (Table 2, 79.71% nationwide). Pit latrines are the next most common toilet in most regions [128]. The majority of the pit latrines in use are not slabbed and therefore not considered improved and sufficiently safe to protect against waste spillage. Still, basic pit latrines should be able to contain most domestic fecal wastes from the open environment [128,131]. However, the lack of an adjacent water source in many basic pit latrines may still drive people to open defecate where water is readily available, such as in rivers. Moreover, >3% of households in most endemic regions have no toilet facility and have no alternative other than to defecate in open fields or waters [128]. In 2022, only 32.32% barangays were certified zero-open defecation (ZOD) communities. Regions with the lowest percentage of ZOD-certified barangays are CALABARZON (6.00%), MIMAROPA (7.81%), and the Davao Region (7.92%), despite a high coverage of households with sanitary toilets in CALABARZON (82.61%) and the Davao Region (90.25%) [128,135]. While ZOD statistics may be due to inconsistent reporting among villages and regions, the data still show high prevalence of open defecation even with access to toilet facilities. Moreover, these numbers do not include excretion in toilet facilities that still drain to the environment, which is also common in low socioeconomic, endemic regions [128]. BARMM sticks as the region with the highest percentage of households with no access to safe WASH, reflecting the extreme poverty in the area [127,128].

## 6. Disease Impact

In 2022, public health facilities recorded 1260 clinically diagnosed acute cases of schistosomiasis and 424 chronic cases with 238 treated in government hospitals [135]; no data on how cases were classified are available. A survey of hospital records in high prevalence provinces found 1415 hospitalization cases due to schistosomiasis in 2013 with disproportionately high cases of severe schistosomiasis—22% hepatic complications, 20% neuroschistosomiasis, a rare form of schistosomiasis where adult worms aberrantly migrate near the central nervous system and deposit eggs that may be transported all the way to the brain, causing seizure and headache [21,145]. These numbers are likely underestimates of the true burden of the disease due to underreporting and scarce access to health facilities [16].

The initial stages and acute form of schistosomiasis are fairly benign, characterized by itchy or bumpy rashes on the site of infection called schistosome or cercarial dermatitis elicited by an allergic response. Katayama syndrome, which occurs 14 to 84 days post infection, may present in naïve acute infections (first schistosome infection) or in heavy reinfections, characterized by sudden onset of fever, malaise, myalgia, headache, eosinophilia, fatigue, and abdominal pain [146,147].

Long-term schistosomiasis as a result of re-infection and insufficient or lack of treatment can lead to the more severe chronic schistosomiasis. Morbidities associated with chronic schistosomiasis are the result of the host inflammatory response to the deposition of parasite eggs in host tissues and organs [148,149]. Venous blood flow carries parasite eggs to the liver and spleen where egg proteases and toxic moieties cause necrosis and initiate an inflammatory response that results in granuloma formation and fibrosis. As more eggs accumulate from persistent infection, liver fibrosis becomes more extensive, leading to the fatal hepatosplenic disease, characterized by portal hypertension, pulmonary hypertension, glomerulopathy, splenomegaly, and thrombocytopenia [118,148]. Egg deposition in the intestine tissues similarly results in the formation of granulomas and eventually fibrosis and hypertrophy of the muscularis mucosa [150]. Symptoms of intestinal schistosomiasis include abdominal pain, altered bowel habits, and bloody stools [148]. These morbidities may persist despite preventative chemotherapy and treatment, as observed in endemic communities in Leyte where at least one hepatosplenic complication was observed in 89.3% subjects despite decade-long morbidity management with praziquantel [38].

Chronic inflammation due to schistosomiasis has also been implicated in subtle morbidities primarily affecting children, namely anemia, stunted growth, and inferior cognition [123,151,152]. Undernutrition is prevalent in these endemic villages, leaving most of the children even more susceptible to parasitic infections and creating a cycle of ill health and poverty [37,130]. Cognitive performance is also affected with chronic infections in children correlated with poor learning, memory, and verbal fluency [152,153]. Low hemoglobin levels associated with schistosomiasis persist even in adult infections [123].

Another complication in endemic areas, is the occurrence of co-infection or polyparasitism with other pathogens such as STHs and intestinal protozoa [154,155,156]. STHs and intestinal parasites often share overlapping endemic regions with schistosomiasis, and there are shared risk factors for infection. Two studies in Palapag from Northern Samar province both showed remarkably high polyparasitism with most study participants having at least two parasite infections [154,155,156].

## 7. Current Philippine Schistosomiasis Control Programs

Past national efforts to control schistosomiasis mainly relied on MDA, which has been found to be insufficient and unsustainable, with persistent morbidity in the population [38]. As the Philippines transitions from elimination of schistosomiasis as a public health problem to interruption of transmission, it is increasingly important to adapt integrated control efforts targeting the different aspects of the disease [16,157].

With the goal of schistosomiasis elimination in humans, animals, and snails by 2025, the country’s National Schistosomiasis Control and Elimination Program (NSCEP) developed a 7-year strategic plan starting in 2019 anchored on the One Health approach and multi-sectoral partnership [16]. With a budget of PhP 5.6 billion (USD 100.7 million), the One Health strategy encompasses preventative chemotherapy, treatment, and management of domestic animals and livestock, focal snail control, improved WASH, and health promotion. Additionally, key in the current programs is stronger partnerships of the DOH with other government agencies, mainly the Department of Education (DepEd), Department of Agriculture (DA), National Irrigation Administration (NIA), and Department of Public Works and Highway (DPWH) and local government units, and non-government organizations. Aside from the implementation and expansion of control programs, the strategy also aims for stronger project management and monitoring [16].

### 7.1. Preventative Chemotherapy

The country’s previous MDA strategy starting in 2007 covered endemic populations between the ages of 5 and 65 years for administration of a single dose of 40 mg praziquantel/kg body weight with the dose increased to 60 mg/kg (split dose) for treatment of KK-positive individuals [158]. Lactating mothers were included for MDA but had to withhold breastfeeding 48 h after drug intake. Mothers in the first trimester of pregnancy were excluded despite the WHO regarding pregnant individuals as eligible for praziquantel. The WHO did not recommend MDA of children under 4 years old given the lack of information about the safety of the drug to the age group [158,159]. The MDA strategy in schools and villages enjoined all of the 28 endemic provinces. Identified provinces which reached <1/100,000 prevalence, namely Bohol, Zamboanga del Norte, Davao del Sur, and Surigao del Sur, were only directed to perform MDA among school children [160].

The 2019 strategy [16] limited MDA to highly endemic barangays covering residents between the ages of 5 and 65 years. The plan shifts to selective treatment of confirmed cases for moderate- and low-prevalence barangays with active case findings in the former, while only patients “seeking consult in the health facility if found positive” are treated in the latter. MDA of schoolchildren has traditionally been conducted in schools, mostly public ones in collaboration with the DepEd, while community-based MDA activities are conducted by municipal- or city-level health offices to their respective endemic barangays [16].

Despite harmonization with MDA activities of other NTDs, the nationwide coverage remains abysmal, annually failing to reach the department’s target of ≥85%. From 2009 to 2021, the highest coverage was achieved in 2017 at 65%. Several reasons have been cited for the low compliance, such as MDA fatigue, fear of adverse reactions from the drug, religious beliefs, and the unappealing taste and size of the drug. Logistic setbacks were also encountered, including delayed procurement and delivery of drugs resulting in some orders left unused and near expiration [16,161]. The COVID-19 pandemic further undermined the MDA efforts, with many such activities postponed and funds and staff redirected to the pandemic response [162].

While the national coverage leaves much to be desired, a number of barangays and provinces consistently attained more that 85% MDA coverage through integrating activities that encourage participation such as distribution of groceries and conduct of small group lectures. Presuming that the current MDA activities still exclude pregnant women, expansion to this vulnerable sector should be explored in light of more evidence demonstrating the safety of praziquantel with no significant effect on birthweight and safety outcomes in a field trial in Leyte [163]. As early as 2002, the WHO recommended the inclusion of pregnant and lactating women for treatment based on human and experimental animal toxicity data and the high risk (anemia, development of chronic infection) of untreated schistosomiasis in the group [164]. Moreover, a field trial of praziquantel testing its safety and efficacy in preschoolers has been initiated [165]. Different dosing may also be explored in light of a study showing that a split dose resulted in significantly more reduced prevalence and intensity than one dose [166]. Studies show contradicting results about the efficacy of 40 mg/kg dose—cure rate of 52% (vs 91% for 60–100 mg/kg divided into at least 2 doses) in one study [167], and an efficacy of 90% based on egg reduction in another Philippine cohort [168].

### 7.2. Testing and Disease Management

Schistosomiasis testing using the KK technique is directed to be available in health facilities starting at the municipal or city level in rural health units (RHU). At the barangay level, barangay health stations (BHSs) can administer rapid diagnostic tests if available. Moreover, serological testing is put forth as an additional test in moderate- to low-endemicity communities to overcome the diminished sensitivity of the KK method in low-intensity cases. Positive cases are then referred to public hospitals for management and treatment [16].

While 100% of public hospitals and RHUs in endemic areas are reported to be capable of managing and treating schistosomiasis cases, testing capacity and competency remain limited. The availability of focal survey data in 90% of endemic barangays suggests that the majority of the endemic populations have access to schistosomiasis testing. Crucial to these laboratories are quality assessments and trainings of more medical technologists given the cited shortage of staff in health units [16,169].

The DOH Research Institute for Tropical Medicine (RITM), the county’s research center for NTDs, has been conducting competency-based trainings on NTD testing with medical technologists with six endemic regions and one non-endemic region trained in the KK technique in 2019 [169]. This training role has recently been institutionalized with the establishment of the National Reference Laboratory (NRL) for Schistosomiasis in RITM. The reference laboratory is also aimed to perform a quality assessment of schistosomiasis laboratories and diagnostics, and to conduct research towards the development of more sensitive and field-applicable tests [170].

### 7.3. Animal Treatment and Management

A major gap in the past control program is the lack of policy on animal treatment and management. With this, central to the current control of animal schistosomiasis is the collaboration with the Department of Agriculture and Bureau of Animal Industry starting with a demonstration project in Northern Samar that began in 2017, which will inform and originate the development of national policies on control, diagnosis, management, and treatment of animal (buffalo, cattle, dogs, sheep, pigs, and goats) schistosomiasis [16]. The strategy entails the establishment of an animal schistosomiasis laboratory in every endemic province that is responsible for testing of animal samples and the training of provincial and municipal veterinarians and livestock technicians to respond in animal deworming and treatment in barangays. As of 2018, 6 of 28 endemic provinces have an established animal schistosomiasis laboratory [16].

### 7.4. Environmental Controls

The national strategy also encompasses environmental and engineering controls that target snail habitats. Past efforts mainly relied on regular clearings of potential snail habitats such as canals, rivers, and wells [16]. Some environmental modifications have also been implemented, including cementing of pathways and earth filling [16,55,171], and infrastructures constructed such as canals and footbridges, that minimize exposure to potentially contaminated waters, with the National Irrigation Administration (NIA) and Department of Public Works and Highway (DPWH) working as key collaborating agencies. The current strategy also explores changes to farming such as crop diversification, modern farming methods, and aqua-culture diversification [16].

Infrastructures that expand access to improved and safe WASH and facilitate proper animal waste management are also beneficial for discouraging and curtailing environmental contamination of human and animal feces. Some LGUs facilitated the construction of communal toilets and a past effort cited entering written agreements with households to construct residential toilet facilities. In the current strategy, WASH infrastructures and environmental modifications are allocated funds from the DOH and local government budgets [16].

Requisite and crucial to these environmental controls are extensive mapping and assessment of snail habitats with trained malacologists to determine appropriate engineering measures for each barangay. Challenges to these efforts are environmental factors such as dams, floods, deforestation, and global warming, which may modify snail habitats and schistosomiasis transmission patterns [6].

### 7.5. Health Education

Recognizing the role of human behavior in the persistence of schistosomiasis, health education acts to promote the correct knowledge, attitudes, and practices (KAP) of schistosomiasis in endemic populations. Slated health education programs include WASH with Health Education (WASHED) to promote sanitary practices and deter open defecation and swimming in potentially contaminated freshwaters, as well as promote responsible animal ownership, including animal waste disposal [16].

A survey in an endemic barangay in Northern Leyte showed that the majority of participants had high awareness of correct KAP, but this was inconsistent with actual behaviors, with participants performing regular water contact activities without protective gear. The majority of water contact activities are occupational rather than recreational [172]. To address this, the development of an intervention package specifically targeting vulnerable occupations (farmers, irrigation workers, and miners) is underway [16].

Water contact measures generated through questionnaires can be used to assess individual practices, allowing for more targeted health education and public intervention. However, precaution should be taken in interpreting these results and efforts should be taken to generate questionnaires that better reflect observed water contact [173].

Belief that schistosomiasis is preventable is associated with higher receptiveness to participating in control program activities such as carabao vaccination [172]. In Leyte, Francisco et al. determined that the high awareness of the severity of schistosomiasis led to higher willingness to participate in MDA [172]. However, while knowledge and awareness are high, they do not translate to disease prevention behaviors possibly as they are deemed not practical in daily work.

### 7.6. Surveillance

To monitor the status of schistosomiasis and progress of control efforts, the DOH instituted annual sentinel surveillance of Grade 3 students (~8 years old) in public schools, and snail surveys every three years are in place with plans for annual snail and animal surveillance. Moreover, regular reporting of cases is directed with monthly to quarterly reporting for high prevalence barangays [16]. Using the geographic information system as a tool for schistosomiasis surveillance may be explored [174].

## 8. Future and Prospect of Schistosomiasis Elimination in the Philippines

The current national control strategy has less than a year left to achieve the target of complete schistosomiasis interruption of transmission by 2025. The goal is ambitious yet admirable, but will be unlikely to be achieved in the foreseeable future. The strategy is also hampered by many challenges, the biggest being the COVID-19 pandemic that persists today. Other challenges include climate change and eco-tourism, which threaten the spread of schistosomiasis [64,89,90]. Optimistically, the programs lay the foundation for an integrated, holistic, multi-faceted, and multi-sectoral control of schistosomiasis distribution and transmission. With the current decisive shift to integrated control and the headway made in the different aspects of schistosomiasis control, the prospect of disease elimination in the Philippines is promising.

As disease prevalence and infection intensity abate, it is crucial that case finding and surveillance activities use more sensitive and specific, but still field-applicable, diagnostics than the currently used KK. Molecular- and antibody-based POC tests are ideal for field detection of current, low-intensity infections, and several advancements have been achieved in *S. japonicum* POC development [175,176,177,178].

The current strategy also expanded testing, treatment, and management to animal schistosomiasis, primarily in carabao. The long-term and more sustainable goal would be the reduction in or replacement of carabaos in land tilling through mechanization as has been achieved in parts of China. Currently, the progress of mechanized farming in the country remains low and slow, although many farmers are amenable to the prospect of higher profit. Where removal of carabao may not be feasible, vaccination would be the next best option. Currently, there have been two main candidate vaccines for carabao that have undergone trials: the *S. japonicum* paramyosin (Sj97) recombinant protein vaccine was shown to be safe, with ≥50% reduction in worm burden in three field trials and has been recommended for a phase II trial [179], and the DNA vaccine SjCTPI fused to bovine heat shock protein 70, which reached a phase III trial in Northern Samar, showing that bovine vaccination combined with snail mollusciciding resulted in 31% reduction in human infections [180]. The progress in schistosome transcriptomics, proteomics, immunomics and exosomics, and mRNA vaccine technology should accelerate vaccine discovery and testing, making the reality of a publicly available licensed vaccine closer than ever [181,182]. Attention should also be given to other livestock and domestic animals and limit free-roaming animals with fenced husbandry. In the absence of bovines, these other free-roaming animals may serve as alternative reservoir hosts [113,141].

Other reforms in agriculture and irrigation, such as paddy-upland rotation and concrete lining of ditches and canals, as is carried out in China, should complement the usual practice of clearing and filling of snail habitats. Even more ecologically and economically sustainable practices, as are used in China, are agroforestry and planting of fast-growing trees in endemic snail sites, which resulted in reduction in snail density to zero after a decade by changing the environment diversity and microclimates (soil temperature, humidity, pH, vegetation density, water table, and light conditions) [141,183]. Trees and herbaceous plants with molluscicide properties are also better snail control than chemical molluscicide (niclosamide) and the introduction of snail predators, such as the red swamp crayfish (*Procambarus clarkii*), which are environmentally disruptive [66]. In addition, the application of snail gene editing to drive suppression or replacement of original susceptible snail population is being explored [184].

Finally, the success of these control programs relies on the households in these endemic communities as the main drivers and grassroot actors in these interventions. People are knowledgeable about schistosomiasis and its prevention as shown in Leyte, although other endemic communities, such as in Mindanao, where there are less schistosomiasis research activities, are potentially less informed. Knowledge of prevention, however, does not exactly translate to behavior. Socioeconomic features are major complicating factors affecting human behavior. Schistosomiasis is the least of concern in many poor schistosomiasis endemic communities where agriculture is the main livelihood and access to safe WASH is scarce. In some areas, the chronic vulnerability to destructive typhoons and other natural disasters and armed conflicts are more pressing matters.

Schistosomiasis has become a feature of poverty in the Philippines, and it may only be completely eradicated once these communities are lifted from poverty. Ultimately, economic reforms towards equitable progress and development across the archipelago of the Philippines are the cures to the sicknesses that are poverty and schistosomiasis.

## Data Availability

No new data were created or analyzed in this study. Data sharing is not applicable to this article.

## Supplementary Materials

The following supporting information can be downloaded at: https://www.mdpi.com/article/10.3390/tropicalmed10020029/s1, Table S1: Human schistosomiasis national prevalence and village level focal surveys in the Philippines [16,29,30,31,51]. Table S2: Human schistosomiasis prevalence in endemic areas based on various diagnostics tests [36,37,38,39,40,41,43,44,50,63,185,186].
