# Supplementary material for: Schistosomiasis in the Philippines: A Comprehensive Review of Epidemiology and Current Control
Source: Trop Med Infect Dis. 2025 Jan 21;10(2):29. doi: 10.3390/tropicalmed10020029 (PMC11860700; doi:10.3390/tropicalmed10020029)
Supplement: Supplementary file 1 [file tropicalmed-10-00029-s001.zip › tropicalmed-3309208-supplementary.pdf]

Table S1. Human schistosomiasis national prevalence and village-level focal surveys* in the Philippines.

Geography of endemic areas			National prevalence (%)			Village-level focal surveys [16]							
							# of endemic barangays						
Island Group	Region	Province	1995 [51]	2005-2008 [31] [30] 2 years and above	2013-2015 [29] 5 to 16 years old	Prevalence (%)	total	0%	low	moderate	high	not surveyed	Coverage
Luzon	Cagayan Valley	Cagayan	--	0.4	2.4	6.6	5	1	1	2	1	0	100%
	Mimaropa	Oriental Mindoro	2.27	6.3	0.2	9.5	33	0	1	13	18	1	97%
	Bicol Region	Sorsogon	3.94	3.6	4	4.9	15	0	0	8	7	0	100%
Visayas	Western Visayas	Negros Occidental	--	0	0.3	0.3	3	2	1	0	0	0	100%
	Central Visayas	Bohol	0.06	0	0	no data	8	0	0	0	0	8	0%
	Eastern Visayas	Eastern Samar	2.91	1.8	0.2	4.2	219	82	17	38	48	34	84%
		Leyte	2.65	0.9	0.5	8.1	345	26	16	87	184	32	91%
		Northern Samar	10.11	2.4	10	6.2	167	3	19	60	81	4	98%
		Samar (Western Samar)	4.21	0.8	1.4	5.4	142	28	11	54	49	0	100%
Mindanao	Zamboanga Peninsula	Zamboanga del Norte	0.06	--	0.2	0.04	9	0	8	1	0	0	100%
		Zamboanga del Sur	~5.85	0	0.3	no data	64	0	0	0	0	64	0%
		Zamboanga Sibugay		0	1.3	0.04	8	0	0	0	0	8	0%
	Northern Mindanao	Bukidnon	1.88	1.8	3.8	2.1	54	1	15	30	8	0	100%
		Lanao del Norte	4.46	0.8	1.2	8.2	41	0	0	10	31	0	100%
		Misamis Occidental	1	0	0.6	0.9	17	5	3	8	1	0	100%
	Davao Region	Davao de Oro (Compostela Valley)	~6.88	0.7	0	0.9	58	13	29	14	2	0	100%
		Davao del Norte		0.8	0.6	0.8	37	13	14	10	0	0	100%
		Davao del Sur	1.29	0.1	3	0	14	14	0	0	0	0	100%
		Davao Oriental	0	--	0.4	0.3	1	0	1	0	0	0	100%
	SOCCSKARGEN	Cotabato (Northern Cotabato)	1.05	0.5	3.5	1.7	17	0	0	0	0	17	0%
		South Cotabato	0.85	0.3	0.4	0.1	16	0	15	1	0	0	100%

		Sultan Kudarat	6.68	0.2	1	2.4	12	0	0	0	0	12	0%
	Caraga	Agusan del Norte	6.88	0.1	4.3	0.3	64	6	9	5	0	44	31%
		Agusan del Sur	7.31	3.9	3.2	3.1	141	18	13	33	20	57	60%
		Surigao del Norte	13.87	0.3	1.5	2	65	8	11	6	3	37	43%
		Surigao del Sur	3.34	1.3	0.4	0.2	28	8	3	1	0	16	43%
	BARMM	Lanao del Sur	4.26	0.4	3	no data	12	0	0	0	0	12	0%
		Maguindanao del Norte & Maguindanao del Sur	21.69	1.8	--	0.1	16	14	1	1	0	0	100%
**Overall**			**4.5%**	**2%**	**0.8-1%**		**1611**	**242**	**188**	**382**	**453**	**346**	

*focal surveys are conducted in endemic villages only and estimate village-level prevalence. National prevalence surveys cover endemic and non-endemic provinces and randomly select villages to be surveyed to estimate province level prevalence.

Table S2. Human schistosomiasis prevalence in endemic areas based on various diagnostics tests.

Endemic areas			Prevalence (%)				Reference
**Province**	**Municipality**	**Barangay**	**KK**	**Molecular**	**Immuno**	**US**	
Sorsogon	Irosin	Bagsangan	8.89*	31.11	66.67	46.67	[43]
Eastern Samar	Oras	Binalayan	--	--	89	--	[185]
		Mabuhay	--	--		--	
		Saugan	--	--		--	
		Taguib	--	--		--	
Leyte	Alang-alang	Bugho	28.3***	--	53.5	28.9	[41]
		SAF	26.6***	--	49.6	16.5	
	Palo	Cangumbang	20.3***	--	30.4	27.5	
		Tacurangan	32.9***	--	57.5	12.3	
	Julita	Dita	35.4***	--	41.8	27.5	
		Calibasag	25.4***	--	27.2	27.2	
	Santa Fe	San Juan	18.5***	--	28.4	12.3	
		San Roque	27.4***	--	41.6	23.7	
Northern Samar	Palapag	6 barangays	22.9**	90.2	--	--	[39]
	Palapag, Laoang	18 barangays	26.2**	25.5-74.5	12.4^, 24.5-66	--	[40, 50, 186]
		22 barangays	27.1**	--	--	--	[38]
		5 barangays	20.1**	--	--	--	[37, 63]
Western Samar	San Jorge	Cantaguic	30.8**	75.0-92.3	--	--	[36]
	Gandara	Casabahan	--	--	78.3	--	[185]
		Conception	--	--		--	
		Nacube	--	--		--	
		San Agustin	--	--		--	
Surigao del Norte	Burgos	Poblacion	3.1*	1.6-27.1	48.5	--	[44]
		San Mateo				--	
	Del Carmen	Cancohoy	11.1*		77.5	--	
		Jamoyaon				--	
	Pilar	Caridad	1.3*		51.4	--	
		San Roque				--	
	San Isidro	Buhing	2.6*		84.8	--	
		Kalipay				--	
		Del Pilar				--	
		Sta. Nino				--	
		Sta. Paz				--	
	Santa Monica	Libertad	1.1*		50.6	--	

*1-stook Kato-Katz (KK) **2-stook KK ***3-stool KK ^ point-of-care circulating cathodic antigen (POC-CCA)
